# Assessing Documentation of Analgesic Prescribing in a Medium Secure Forensic Psychiatric Setting

**DOI:** 10.1192/bjo.2025.10084

**Published:** 2025-06-20

**Authors:** Bethan Bown, Robert Stamatakis

**Affiliations:** 1 Cardiff University, Cardiff, United Kingdom; 2 Swansea Bay University Health Board, Swansea, United Kingdom

## Abstract

**Aims:** This service evaluation sought to assess the consistency of documentation in 5 key areas of analgesic prescribing in a medium secure forensic unit in South Wales.

**Methods:** Five key areas which are important to document when prescribing analgesia were defined as follows: 1) Indication, 2) Prescription Review, 3) Risk, 4) Discontinuation Guidance and 5) Patient Counselling on Analgesic Choice. Data was collated on these 5 key areas for opioid and pregabalin prescriptions between 1 November 2023 and 1 April 2024. Using Hospital Electronic Prescribing and Medicines Administration (HEPMA), it was possible to establish prescription data. Information on each prescription was then collated from: clinical team meeting (CTM) notes, nursing notes, GP contact records and tribunal reports for each patient.

**Results:** There were 18 analgesic prescriptions which fitted project criteria. 11% prescriptions were for morphine, 17% for co-codamol, 39% for codeine and 33% for pregabalin. Documentation across the 5 key areas was deficient, with 0% patients with documentation in all 5 key areas, 14% patients with documentation in 4 areas, 36% patients with documentation in 3 areas and 50% patients with documentation in <2 areas. Indications were better recorded in CTM notes than on HEPMA. On HEPMA, only 50% prescriptions had an indication, and of those only 6% had a specific indication with the remainder noted as “pain” (33%) or “pain team advice” (11%). In comparison, 90% prescriptions from CTM notes had an indication; the most common indication being leg pain (40%). In terms of prescription reviews, only 56% prescriptions were reviewed. No patients had any documented consideration of the risk of prescribing analgesia based on their substance misuse history despite 93% patients included having a recorded substance misuse history. 57% patients were prescribed the drug they have a recorded history of addiction to. Only 36% prescriptions documented the physical health risks of prescribing analgesia. Similarly, there was no documented guidance for any patient on circumstances to discontinue analgesia. In regard to patient counselling, only 50% patients were counselled on the choice of analgesia.

**Conclusion:** Multiple sources of information made it time consuming to get a holistic view of each prescription. Some of the key areas such as discontinuation guidance and substance misuse risk were not documented at all, with other areas having sporadic documentation depending on the prescriber. To improve future practice, changing HEPMA to have mandatory fields to record 5 key areas when prescribing analgesia would ensure consistency of documentation.

